# The efficacy of community-based exercise programs on circulating irisin level, muscle strength, cardiorespiratory endurance, and body composition for ischemic stroke: a randomized controlled trial

**DOI:** 10.3389/fneur.2023.1187666

**Published:** 2023-06-29

**Authors:** Dongheon Kang, Jiyoung Park, Seon-Deok Eun

**Affiliations:** Department of Healthcare and Public Health Research, National Rehabilitation Center, Ministry of Health and Welfare, Seoul, Republic of Korea

**Keywords:** stroke, irisin, muscle strength, cardiorespiratory fitness, disability

## Abstract

**Objective:**

We investigated the changes in circulating irisin levels after community-based exercise and the association of these levels with improvements in muscle strength, cardiorespiratory endurance, and body composition in people with ischemic stroke.

**Methods:**

Twenty participants were randomly assigned to either a control or an exercise group. The community-based exercise program (CEP) consisted of 8 weeks of 1 h sessions for 3 days a week. Irisin levels, muscle strength, cardiorespiratory endurance, and body composition were assessed before and after the intervention.

**Results:**

Significant improvements were observed in the leg and trunk strength, peak oxygen consumption values, and body composition of the exercise group compared with the control group. In addition, circulating irisin levels were observed to have increased in the exercise group, positively correlated with muscle strength and cardiorespiratory endurance.

**Conclusion:**

CEP might be an effective intervention to increase irisin levels and prevent a stroke-related decline in muscle function.

## 1. Introduction

The global incidence of stroke has increased over the past few decades, making it one of the leading causes of death and long-term disability (1, 2). Stroke is the second and third leading cause of death worldwide and in South Korea (after cancer and cardiovascular disease), respectively (3, 4), resulting in a significant economic burden for both patients and society (5). Ischemic stroke (IS) accounts for 80% of stroke occurrences (6). Inadequate blood flow via brain vessels leads to impaired glucose supply, cerebral hypoxia, and impaired elimination of superfluous metabolites, ultimately leading to cerebral infarction (7).

Despite various advances in diagnostic and therapeutic procedures, IS remains one of the leading causes of long-term disability and death (8). Controlling disease-causing risk factors and improving disease prediction are critical methods to minimize the rate of disability in patients with IS. Rapidly measurable biomarkers of stroke functional outcomes and mortality benefit optimal care and healthcare resource allocation (9).

Irisin, a newly discovered myokine peptide (10), is a cleaved and secreted fragment of the fibronectin type III domain-containing protein 5 (FNDC5) and was initially documented as a new hormone secreted from muscle cells during exercise. The highest basal level expression of *FNDC5* is observed in the brain and heart (11). It is encoded by *PGC-1α*, which is involved in many pathways associated with energy metabolism (10).

Various animal experiments and clinical studies have suggested that irisin plays a neuroprotective role through different biological mechanisms and improves the neurological function of people with IS. A previous study found that serum irisin concentrations decreased following IS (12). Moreover, irisin levels were inversely related to cerebral infarction volume, late neurological impairment, and IL-6 and TNF-α concentrations. Administration of recombinant irisin to diabetic mice decreased the size of cerebral infarcts and neurological deficits.

A previously published multicenter study examined the correlation between irisin concentration and the prognosis of IS with a large sample size (12). This study revealed that a lower irisin concentration was associated with poor functional prognosis in patients with IS. Tu et al. also discovered in a study involving 324 people that low serum irisin levels predicted poor early functional outcomes in IS (12). These findings suggest that irisin may be associated with stroke risk and functional recovery following a stroke. People who have had a stroke require rehabilitation to overcome functional disabilities. Accordingly, interest in exercise-based rehabilitation is increasing (13).

Exercise-based rehabilitation effectively improves physical fitness, cardiopulmonary endurance, and muscle strength, particularly in people with stroke (14). However, the effect of exercise training, while taking into account intensity (high-or moderate-intensity exercise), type (strength or cardiorespiratory endurance exercise), and duration (acute or chronic exercise), on circulating and muscle irisin expression is controversial. Muscle strength and cardiorespiratory endurance training were considered effective interventions in improving stroke-related problems; however, the effect of muscle strength and cardiorespiratory endurance training on irisin expression in the stroke population is yet to be reported. Therefore, in this study, we aimed to determine the changes in circulating irisin levels and the association of irisin levels with improvements in muscle strength, cardiorespiratory endurance, and body composition following community-based exercise.

## 2. Materials and methods

### 2.1. Participants and study design

Twenty volunteers recruited from the community health and outpatient stroke- rehabilitation centers were assessed for eligibility. Our research group, independent of the rest of the study staff, randomly selected participants who met the inclusion criteria (*n* = 20) before the intervention using a computer-generated allocation schedule. Each participant had to draw a sealed envelope and was randomly assigned to either the experimental (EX) (*n* = 10) or the control (*n* = 10) group. Four subjects dropped out of the program, leaving 16 participants (six and 10 in the EX and control groups, respectively). The inclusion criteria were as follows: (1) participants diagnosed with IS; (2) Mini-Mental State Examination score > 22; (3) ability to follow verbal instruction and communicate; (4) ability to understand the study’s procedure and purpose; and (5) Voluntary participation. The exclusion criteria were as follows: (1) those with neurological disorders other than stroke (e.g., Parkinson’s disease); (2) those with a hip or lower extremity pressure ulcer; (3) those with orthopedic problems in the lower extremities; and (4) those with other cardiopulmonary diseases or those who could not perform tests due to musculoskeletal problems. This study was approved by the National Rehabilitation Hospital Institutional Review Board (NRC-2015-02-014). The study protocol was registered and assigned KCT0005782 (first registration 01/07/2021). Written informed consent was obtained from all participants, and the study conformed to the Declaration of Helsinki guidelines.

### 2.2. Data collection

#### 2.2.1. Irisin

Blood samples to test for irisin were drawn by trained nurses between 07:30 h and 10:00 h after 12 h of overnight fasting and 2 days of minimal physical activity. Samples were centrifuged and stored at −80°C. Irisin levels were determined using commercial enzyme-linked immunosorbent assay (ELISA) kits following the manufacturer’s instructions (Catalog no. EK-067-52, Phoenix, AZ, United States). The detectable range of the irisin ELISA kit was 0.066–1024 ng/mL.

#### 2.2.2. Muscle strength

An isometric dynamometer (HUR, Kokkola, Finland) was used to measure the strength of the lower limbs. An isometric contraction test was performed to evaluate the knee extension/flexion and trunk extension/flexion. In knee extension/flexion cases, the non-affected and affected sides were measured separately for stroke. This test measured the maximum muscle strength when the muscle contracted without moving against a fixed object. Before testing, participants were asked to perform two regular practices for the test, and the average values of the three recorded measurements were used in the analysis.

#### 2.2.3. Cardiorespiratory endurance

To determine cardiorespiratory endurance, such as peak oxygen uptake (VO_2_ peak), each participant performed a graded exercise test on the cycle ergometer (Angio CPET with static wall fixation, Lode B.V., Groningen, Netherlands). Considering the physical fitness level of the patients, the protocol for the graded exercise test on the cycle ergometer was set to maintain 60 rpm (15). Warm-up exercises were performed at 0 W for the first 2 min, and the workload gradually increased by 10 W every 1 min. The test was continued until the participants stopped of their own volition due to exhaustion.

The 6-min walk test (6MWT), a sub-maximal exercise used to evaluate aerobic capacity and endurance, was used to measure the total distance walked by the participants in 6 min (16). The walking speed and resting time during the test were adjusted depending on the participant’s ability. The total distance of the walk was recorded as meters walked in 6 min.

#### 2.2.4. Body composition

Body composition measurements were performed before and after the exercise training. The height was evaluated using an extensometer. Body weight, body mass index (BMI), skeletal muscle mass, and body fat were measured by bioimpedance analysis using the Inbody S10 (Inbody, Seoul, South Korea).

### 2.3. Community-based exercise program

A community-based exercise program (CEP) was conducted 3 days a week for 8 weeks at the health-promotion center. The CEP program consisted of 60 min sessions of whole-body exercise. The initial duration of each exercise gradually progressed to a maximum duration of 45 min, based on the individual’s abilities. The CEP was conducted at 65–80% of the individual’s maximum heart rate, measured at the baseline. The participants alternated between three modes of exercise [15 min strength training, 15 min cardiovascular exercise, and 15 min game-based leisure-time physical activities (GLPA) for 8 weeks]. Strength training involved the use of a thera-band (Hygenic Corporation, Akron, OH, United States) for seven exercises for the upper body (shoulder press, seated rows, back row, lat pull down, chest press, biceps curl, and triceps extension) and 10 exercises for the lower body (squat, lunge, deadlift, bridge, back extension, superman position, leg raise, reverse crunch, sit-up, and crunch). The exercise intensity was based on the color of the thera-band, and 15 RM of the lateral raise using the thera-band was performed. The subjects were asked to perform 12–15 repetitions of three sets for all upper and lower body exercises. Each exercise was performed using the thera-band in the power training protocol (17), with a concentric contraction phase as soon as possible, a 1 s pause, and an eccentric contraction phase exceeding 2 s. The cardiovascular exercises were conducted with seven workouts (high knee, sidestep, pogo jump, jumping jack, front step, back step, and knee up) that could be achieved without a machine. The GLPA was conducted to promote participants’ physical function and make the training enjoyable. It consisted of four games, with one game per session.

### 2.4. Statistical analysis

Outcome measures were analyzed in all participants and each group using SPSS 21.0. (IBM SPSS Inc., Chicago, IL, United States). The mean and standard deviation of each variable were obtained using descriptive statistics. The number of samples (n) was <30, and the normality test was performed using the Shapiro–Wilk test; however, the nonparametric test was conducted because it did not satisfy the significance level (*p* < 0.05). Wilcoxon’s signed-rank test was performed to determine any difference between the pre- and post-test results of experimental and control groups. The Mann–Whitney *U*-Test was performed to evaluate the differences in circulating irisin, muscle strength, cardiorespiratory endurance, and body composition between the two groups. The differences between the final and the basal level of variables were indicated as delta. A Spearman rank correlation analysis was performed to evaluate the correlation among the delta values. In addition to this null hypothesis testing, the data were assessed for clinical significance using an approach based on the magnitudes of change. We calculated the magnitude of the size of differences by the effect size (ES) (18). We considered an ES of 0.00–0.19 trivial, 0.20–0.49 small, 0.50–0.79 moderate, and ≥ 0.80 high (18).

## 3. Results

### 3.1. Participant characteristics

The baseline characteristics of the participants, including age, height, weight, skeletal muscle mass, body fat, BMI, sex, location of the stroke, stroke onset, and cognitive status, are shown in Table 1. The mean age of the participants was 55.5 ± 12.93 years, and their average BMI was 24.14 ± 2.96 kg/m^2^. The body composition parameters of the participants mainly included fat- and muscle-related parameters. There were no significant differences in the baseline anthropometric variables between the groups.

**Table 1:** Participants’ characteristics.

Variable	EX	CON	*p*-value
Age, years	54.33 ± 18.22	56.20 ± 9.64	0.958
Height, cm	159.93 ± 7.85	161.87 ± 8.05	0.562
Weight, kg	60.67 ± 5.23	63.78 ± 8.90	0.313
Skeletal muscle mass, kg	23.55 ± 3.03	23.56 ± 4.96	0.875
Body fat, %	28.33 ± 6.50	27.07 ± 8.41	0.368
BMI, kg/m^2^	23.73 ± 1.75	24.39 ± 3.56	0.562
Sex (male/female), %	50/50	70/30	
Location of stroke			
Right/left, %	66.67/33.33	50.00/50.00	
Stroke onset, years	7.96 ± 8.39	5.11 ± 5.18	0.713
Cognitive status, MMSE score	26.83 ± 1.94	26.30 ± 3.23	0.958

EX, experimental group; CON, control group; BMI, body mass index; MMSE, Mini-Mental State Examination. Values are mean ± standard deviation or n (%).

### 3.2. Outcome measures

The changes in irisin, muscle strength, cardiorespiratory endurance, and body composition before and after the program are presented in Table 2. For the EX group, significant improvements were noted in irisin, muscle strength [knee extension/flexion (non-paretic [NP] leg), knee flexion (paretic [P] leg), and trunk extension/flexion], cardiorespiratory endurance (VO_2_ peak, 6MWT), and body composition (skeletal muscle mass) (Table 2).

**Table 2:** Before–after intervention values and changes in the outcome measures according to the groups.

Measurement	Before intervention	After intervention	Gap (change)	Effect size	*Z*	*p*-value
Irisin (ng/mL)						
EX	6.20 ± 1.08	6.77 ± 1.36^*^	0.57 (9.19)	0.46^#^	−2.201	0.028
CON	6.33 ± 1.66	6.16 ± 1.14	−0.17 (−2.69)	0.12	−0.051	0.959
Knee extension (NP) (kg)						
EX	25.15 ± 8.59	32.47 ± 10.22^*^	7.32 (29.11)	0.78^##^	−2.201	0.028
CON	20.76 ± 10.61	19.70 ± 5.96	−1.06 (−5.11)	0.12	−0.357	0.721
Knee flexion (NP) (kg)						
EX	9.58 ± 3.92	14.58 ± 6.38^*^	5.00 (52.19)	0.94^###^	−2.201	0.028
CON	10.42 ± 4.82	9.68 ± 4.85	−0.74 (−7.10)	0.15	−1.274	0.203
Knee extension (P) (kg)						
EX	13.60 ± 9.06	19.49 ± 12.08	5.89 (43.31)	0.55^##^	−1.782	0.075
CON	15.82 ± 11.57	14.59 ± 4.61	−1.23 (−7.77)	0.14	−0.153	0.878
Knee flexion (P) (kg)						
EX	2.85 ± 2.49	8.23 ± 6.34^*^	5.38 (188.77)	1.12^###^	−1.992	0.046
CON	6.65 ± 5.23	5.29 ± 3.87	−1.36 (−20.45)	0.30^#^	−0.663	0.508
Trunk extension (kg)						
EX	11.02 ± 6.78	23.26 ± 12.65^*^	12.24 (111.07)	1.21^###^	−2.201	0.028
CON	13.42 ± 5.41	10.86 ± 6.70	−2.56 (−19.08)	0.42^#^	−1.376	0.169
Trunk flexion (kg)						
EX	10.22 ± 5.06	14.43 ± 6.08^*^	4.21 (41.19)	0.75^##^	−2.201	0.028
CON	11.13 ± 6.67	8.79 ± 6.34^*^	−2.34 (−21.02)	0.36^#^	−2.397	0.017
VO_2_ peak (mL/min/kg)						
EX	14.05 ± 6.47	19.29 ± 7.64^*^	5.24 (37.30)	0.74^##^	−2.201	0.028
CON	15.80 ± 3.37	14.53 ± 3.35^*^	−1.27 (−8.04)	0.37^#^	−2.395	0.017
6MWT (m)						
EX	176.34 ± 125.47	229.05 ± 136.02^*^	52.71 (29.89)	0.40^#^	−2.201	0.028
CON	258.66 ± 72.90	252.24 ± 110.49	−6.42 (−2.48)	0.07	−0.255	0.799
Skeletal muscle mass (kg)						
EX	23.55 ± 3.03	25.50 ± 3.34^*^	1.95 (8.28)	0.61^##^	−2.201	0.028
CON	23.56 ± 4.96	23.11 ± 4.09	−0.45 (−1.91)	0.10	−0.771	0.441
Body fat (%)						
EX	28.33 ± 6.50	24.63 ± 6.63	−3.70 (−13.06)	0.56^##^	−1.782	0.075
CON	27.07 ± 8.41	27.86 ± 6.80	0.79 (2.92)	0.10	−0.510	0.610

EX, experimental group; CON, control group; NP, non-paretic; P, paretic; 6MWT, 6-min walk test. ^#^small; ^##^moderate; ^###^large. ^*^p < 0.05.

**Irisin:** A significant increase was observed in the circulating irisin levels (*p* = 0.028) in the EX group compared with the control group.

**Muscle strength:** Significant improvements were observed in knee extension (NP) (*p* = 0.028), knee flexion (NP) (*p* = 0.028), knee flexion (P) (*p* = 0.046), trunk extension (*p* = 0.028), and trunk flexion (*p* = 0.028) in the EX group compared with the control group.

**Cardiorespiratory endurance:** Significant increases were observed in the VO_2_ peak (*p* = 0.028) and 6MWT (*p* = 0.028) in the EX group compared with the control group.

**Body composition:** Skeletal muscle mass and body fat of the EX group showed a significant increase and decrease, respectively, compared with the control group.

Table 3 shows a correlation analysis with training effects, the association between groups, and the pre–post variations in circulating irisin, muscle strength, and cardiorespiratory endurance. Significant correlations were observed between the group and the pre-post variation for knee flexor (NP) (*R* = 0.756, *p* = 0.001), knee flexor (P) (*R* = 0.672, *p* = 0.004), trunk extension (*R* = 0.784, *p* < 0.001), and trunk flexion (*R* = 0.784, *p* < 0.001) strength; VO_2_ peak (*R* = 0.840, *p* < 0.001); and 6MWT (*R* = 0.504, *p* = 0.046).

**Table 3:** Correlation analysis with training effects, association between groups, and pre–post variations.

Variables		Mann–Whitney *U*-test		Spearman correlation	
		*Z*	*p*-value	*R*	*p*-value
Group	Irisin, ng/mL	−1.085	0.313	0.280	0.293
	Knee extensor (NP), kg	−0.651	0.562	0.168	0.534
	Knee flexor (NP), kg	−2.929	0.002	0.756^**^	0.001
	Knee extensor (P), kg	−1.193	0.263	0.308	0.246
	Knee flexor (P), kg	−2.603	0.007	0.672^**^	0.004
	Trunk extension, kg	−3.037	0.001	0.784^**^	<0.001
	Trunk flexion, kg	−3.039	0.001	0.784^**^	<0.001
	VO_2_ peak, mL/min/kg	−3.254	<0.001	0.840^**^	<0.001
	6MWT, m	−1.952	0.056	0.504^*^	0.046

NP, non-paretic; P, paretic; 6MWT, 6-min walk test. ^*^p < 0.05; ^**^p < 0.01.

Circulating irisin levels were correlated with an increase in skeletal muscle mass (*R* = 0.429) and a decrease in body fat mass (*R* = −0.771) (Table 4).

**Table 4:** Association of circulating irisin levels with skeletal muscle mass and body fat after training.

Variables		*R*
Irisin	Skeletal muscle mass, kg	0.429
	Body fat, %	−0.771

These results suggest that a change in the circulating irisin levels following exercise training was closely related to improving body composition in patients with IS.

## 4. Discussion

In this study, we observed that circulating irisin protein levels increased significantly, with concomitant improvements in muscle strength, cardiorespiratory endurance, and body composition in patients with IS who participated in a community-based exercise program. Furthermore, our research showed that the alternation of circulating irisin was strongly correlated with increased skeletal muscle and decreased body fat. This is the first study to evaluate the effect of community-based exercise on irisin levels and its association with body composition changes for IS in South Korea.

In this study, motivating participants with IS to exercise regularly was essential in achieving high attendance and maximizing exercise effects. Therefore, exercise instructors tried communicating with subjects through text messages and individual consultations to check their daily assignments. All participants expressed satisfaction with the community-based group exercise program.

Strength and cardiovascular training are essential to counter the impairment caused by stroke in muscle strength and cardiorespiratory endurance. This form of training for stroke patients can provide a broad range of systemic benefits, including (19) (1) an increase in muscle strength and prevention of loss of muscle mass; (2) an increase in cardiorespiratory endurance; (3) a decrease in fall risk; and (4) improvements in gait, mobility, and functions. In addition, skeletal muscles are progressively identified as endocrine organs capable of releasing various signaling molecules and regulating myokines, a cytokine that includes irisin. This protein regulates stroke-related pathological and physiological systems. In this study, physical functions, including muscle strength and cardiorespiratory endurance, significantly improved with the increase of the expression of circulating irisin. Therefore, CEP, which includes strength and cardiovascular training, could be a potent intervention for IS to increase muscle strength and cardiorespiratory endurance by improving irisin expression. Exercise intervention stimulates irisin secretion by contracting skeletal muscles (7). Animal experiments and clinical studies have shown that irisin plays a vital role in the nervous system through different biological mechanisms, including a positive effect on preventing neurological diseases and a potential therapeutic effect (20, 21). Higher levels of irisin owing to exercise were shown to protect neurons against ischemia-related damage by activating the Akt and ERK1/2 signaling pathways, significantly reducing the cerebral infarction volume, neuroinflammation, and ischemic oxidative stress. Low serum irisin levels in patients with acute IS may predict the risk of poor functional outcomes (22). In addition, irisin plays a role in the pathogenesis of diseases known to be significant risk factors for cerebrovascular events, including hypertension (23, 24), type 2 diabetes (25), insulin resistance (26), and metabolic syndromes (27). Combined with our findings, exercise-induced irisin may benefit the treatment of stroke-related conditions, particularly neurological diseases, as an endocrine activator of brown fat function.

Increased circulating irisin was previously demonstrated following cardiovascular and strength training (10, 28). Strength training increases strength and muscle fibers (anaerobic), leading to increased levels of myosin protein. Similarly, endurance exercise is hallmarked with fatigue resistance (aerobic), leading to increased mitochondrial proteins (29). Bostrom et al. showed a two-fold improvement in circulating irisin levels in healthy adults compared with a control group after 10 weeks of cardiovascular training (10). Moreover, circulating irisin levels improved immediately after cardiovascular training (30, 31). Kim et al. demonstrated increased circulating irisin levels in older adults who underwent 12 weeks of strength training using an elastic band compared with, a non-training group (28). In addition, circulating irisin levels increased immediately after strength training (32). Haghighi et al. (33) showed that men who were overweight or obese and underwent 8 weeks of cardiovascular and strength training showed a significantly higher irisin serum level and significantly lower body fat percentage and body weight than the control group. In the present study, circulating irisin levels were significantly higher after cardiovascular and strength training in people with IS. Considering the benefits of cardiovascular and strength training in improving muscle strength and cardiorespiratory endurance function for IS, cardiovascular and strength training could be an effective intervention strategy.

This study has several limitations. First, it had a relatively small sample size. Therefore, there is a need to test the beneficial effects of community-based exercise on circulating irisin, including irisin-related mechanisms, in a large randomized sample. Second, this study was performed on participants with IS who could walk. Our results are, therefore, difficult to generalize and apply to all stroke participants. Third, the intervention was limited to 8 weeks. A long-term study accompanied by follow-up tests is recommended to verify the longer-term effect of the program on outcome measures.

## 5. Conclusion

We reveal that community-based exercise can increase the circulating irisin level in participants with IS, with concomitant improvements in muscle strength, cardiorespiratory endurance, and body composition. In addition, there were positive correlations between the increase in circulating irisin levels and the improvements in skeletal muscle mass and body fat. Based on our results and previous reports on the role of irisin in IS, community-based exercise may be an effective intervention strategy to increase circulating irisin levels and positively impact physical fitness.

## Data availability statement

The raw data supporting the conclusions of this article will be made available by the authors, without undue reservation.

## Ethics statement

The studies involving human participants were reviewed and approved by the National Rehabilitation Hospital Institutional Review Board (NRC-2015-02-014). The patients/participants provided their written informed consent to participate in this study.

## Author contributions

DK, JP, and S-DE: conceptualization. DK: methodology and formal analysis. JP: investigation and visualization. DK and JP: data curation and writing—original draft preparation. S-DE: writing—review and editing and funding acquisition. DK and S-DE: project administration. All authors have read and agreed to the published version of the manuscript.

## Funding

This research was funded by the National Rehabilitation Research Institute under grant 15-C-01.

## Conflict of interest

The authors declare that the research was conducted in the absence of any commercial or financial relationships that could be construed as a potential conflict of interest.

## Publisher’s note

All claims expressed in this article are solely those of the authors and do not necessarily represent those of their affiliated organizations, or those of the publisher, the editors and the reviewers. Any product that may be evaluated in this article, or claim that may be made by its manufacturer, is not guaranteed or endorsed by the publisher.

## Abbreviations

6MWT: 6-min walk test
BMI: body mass index
CEP: community-based exercise program
CON: control group
ELISA: enzyme-linked immunosorbent assay
ES: effect size
EX: experimental group
FNDC5: fibronectin type III domain-containing protein 5
GLPA: game-based leisure-time physical activities
IS: ischemic stroke
MMSE: Mini-Mental State Examination
NP: non-paretic
P: paretic
VO2: peak oxygen uptake
